# Black Lives Matter Principles as an Africentric Approach to Improving Black American Health

**DOI:** 10.1007/s40615-020-00845-0

**Published:** 2020-08-13

**Authors:** Kaston D. Anderson-Carpenter

**Affiliations:** Michigan State University, East Lansing, MI USA

**Keywords:** Public health interventions, African American, Black American, Population health, Health disparities, Black Lives Matter

## Abstract

Although public health has made substantial advances in closing the health disparity gap, Black Americans still experience inequalities and inequities. Several theoretical frameworks have been used to develop public health interventions for Black American health; yet the existing paradigms do not fully account for the ontology, epistemology, or axiology of Black American populations. The Black Lives Matter (BLM) movement provides a basis for understanding the constructs that may contribute to Black American health. By drawing from the 13 BLM principles, this paper presents an alternative approach for developing, implementing, and evaluating public health interventions for Black populations in the USA. Furthermore, the approach may inform future public health research and policies to reduce health disparities within and across Black populations in the USA.

*“*When Black people get free, everybody gets free*.”*

~Alicia Garza, co-founder of the Black Lives Matter movement

## Introduction

Despite substantial improvements in reducing the health disparity gap in the USA, Black^1^ Americans still fare far worse than their White peers across almost every health indicator [1, 2]. These disparities often affect Black Americans as early as childhood and extend throughout the lifespan. Not only do Black Americans have a shorter life expectancy relative to their White counterparts [3, 4] but also social and environmental determinants of health have a substantially harmful effect on Black Americans. Even among subgroups of Black American populations, such disparities exist. Black women experience greater decrements in health outcomes such as obesity, diabetes, and cancer compared with women from other racial/ethnic groups [1, 5]. Additionally, Black men who have sex with men (MSM) and Black transgender women have greater odds of housing instability, substance use, and incarceration relative to their heterosexual Black peers [6].

Numerous perspectives have delineated potential mechanisms to explain health disparities in Black American populations. Historically, public health is grounded in a postpositivist epistemology, which has allowed researchers and practitioners to examine the epidemiology of health conditions such as HIV, diabetes, cardiovascular disease, and obesity in Black communities. The ecological systems theory (EST), initially proposed by Bronfenbrenner [7, 8], offers a robust conceptualization of the interacting determinants of public health. Whereas Bronfenbrenner proposed the EST as nested levels (e.g., microsystem is nested in the mesosystem), other scholars [9] have argued that ecological levels are better understood as networked—that is, microsystems interact with one another through a common individual. In public health research and practice, these systems include individual or intrapersonal characteristics, interpersonal processes, institutional and community factors, and public policy. Although the two approaches differ in how ecological levels interact, the implications for addressing health disparities in Black American populations are well-documented [2, 10, 11]. Still, public health research has shown that other models have utility in improving Black American health.

Three such models are the theory of planned behavior, the social determinants of health model, and the health equity measurement framework (HEMF). The theory of planned behavior [12] posits that attitudes, norms, and perceived behavioral control (or skills) interact to influence one’s intention to engage in a health behavior and that both intentions and perceived skills influence health behavior engagement. To understand how knowledge, attitudes, and perceived skills impact behavior, however, one must consider broader ecological factors defined by the social determinants of health (SDH) model [13]. Based on Whitehead’s pioneering work [14], the SDH model, similar to the ecological systems theory, posits that multiple, and often fixed, environmental factors play a role in one’s health behavior and outcomes; such factors often lie outside the control of individuals. Specifically, the SDH model highlights individual factors (e.g., age, gender, sexual orientation, and race/ethnicity); social and community networks (e.g., family involvement and functioning, sense of community); and general socioeconomic, cultural, and environmental conditions (e.g., living and working conditions, racism and discrimination, homophobia, biphobia, and transphobia) that may be outside individual control.

The HEMF [15] built on previous models by introducing a social stratification process. This process highlights the manner in which social hierarchies affect health outcomes through systemically unequal distributions of power, prestige, and resources. The HEMF provides an inclusive process of investigating health equity while integrating multiple frameworks of social determinants of health and identifying potentially strategic points of intervention for diverse populations.

Despite acknowledging that race, gender, and socioeconomic status are important in understanding health inequities, the postpositivist underpinnings of many public health models may prevent them from explicitly demonstrating how Black queer and trans people, older Black adults, and Black women are critical partners—and not merely participants—in understanding health from a Black-centered perspective. The BLM movement differs by affirming those identities; specifically, it asserts those identities as fundamental to understanding Black diversity in the USA. By affirming the identities, the BLM also validates those experiences and realities. Such validation may contribute to developing, implementing, and evaluating public health interventions for Black American health through a critical and intersectional lens. To address some of the limitations of the aforementioned models, I introduce an alternative approach grounded in the Black Lives Matter (BLM) principles and informed by the ecological systems theory, theory of planned behavior, and social determinants of health model for understanding Black health from a critical Africentric epistemology (an expanded discussion on Africentrism can be found elsewhere) [16–19].

### Challenging the Reality of Black Health

**Fig. 1:**
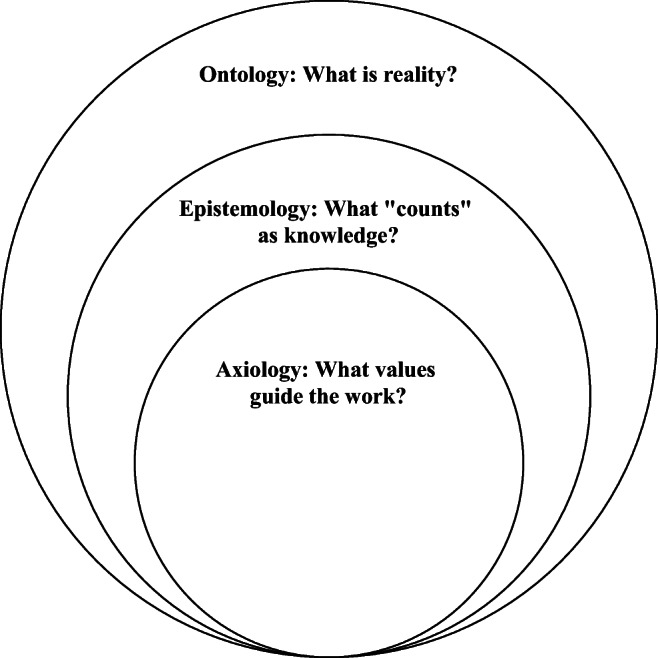
Nested model of philosophical assumptions

Questions of scientific inquiry hinge on ontology, epistemology, and axiology. From these three philosophical assumptions come methods, evaluation procedures, and sustainability processes. To better understand the relationship between ontology, epistemology, and axiology, we can reference the nested structure depicted in Fig. 1. The ontology of public health prompts us to ask, “What is reality?” Scholars have argued that the ontology of public health interprets the “public” as individual and/or collective and “health” as mechanistic and/or dialectic [20]. Other scholars [21] posited that, based on Nijhuis and van der Maesen’s propositions [20], ontological positions that focus on individual health (as opposed to collective health) will produce interventions that less likely to be adopted by communities.

Historically, public health’s ontological positioning has been notably aligned with postpositivism, operating under the assumption that a single reality exists for population health. Much of public health research and practice has been anchored in the notion that there are universal mechanisms that influence health behavior and outcomes in Black populations in the USA and, perhaps, globally. Ontological assumptions guide research in investigating the reality of Black populations experiencing public health outcomes such as HIV risk behavior, substance use, and cardiovascular and metabolic diseases. Although not explicitly stated, ontological positioning in public health research influences the epistemology through which research and practice are implemented.

Epistemology (see Fig. 1, middle sphere) provides the parameters and assumptions that determine what types of evidence “count” as knowledge, what standards are used to define rigorous evidence, and how one obtains knowledge about the reality. Epistemological assumptions provide a framing to discern whether data meet a minimum threshold to be considered evidence. In postpositivism, knowledge is obtained from systematic investigations of phenomena with minimal interaction between the researcher and participant. To be sure, postpositivism is not the sole epistemology in the field. In fact, Whitehead’s pioneering work in health equity [14, 22] helped substantially shift public health language from being deficit oriented to more socially and contextually oriented perspectives. In turn, public health research included more epistemological assumptions to address complex and interlocking contingencies that influence community and population health. Thus, critical epistemologies and theories (e.g., critical race theory, feminist theory, queer theory) have been highlighted as germane to understanding the reality of Black American health.

Whereas many philosophical analyses stop at ontology and epistemology, a catholic conceptualization must also include axiological assumptions (Fig. 1, inner sphere). Axiology is the philosophy of values [23], which may include ethics and social validity [24]. More specifically, axiological matters compel us to ask, “What values do we bring to the area of scientific inquiry?” Following the ontological and epistemological issues highlighted previously, it is incumbent upon public health to evaluate its own values toward Black health. The fundamental question we must ask ourselves is, “What value do we place upon Black lives?” From this question, we can further proceed to reflect upon the following:

1. What are our attitudes and beliefs toward Black Americans?
2. What biases, toward or against, Black people do we bring to scientific inquiry?
3. What is the importance of achieving social validation of goals, procedures, and effects from Black communities in public health research and practice?

By addressing the axiological questions that are specific to Black population health, public health research can strengthen its ethical and philosophical underpinnings in ways that enhance Black health holistically.

Realizing the ontology, epistemology, and axiology of Black health requires a critical analysis of the philosophies that guide intervention development and testing. Although public health research has been guided historically by postpositivism, the field has recognized the need for critical epistemological investigations to understand the ontology of Black American health more fully. However, many existing approaches do not integrate philosophical thoughts and values that are unique to Black populations. However, the BLM movement provides a basis for developing an approach to account for such limitations.

### Why BLM?

Following George Zimmerman’s 2013 acquittal, three Black organizers—Alicia Garza, Patrisse Cullors, and Opal Tometi—mobilized Black communities and their allies to protest the mistreatment of, and disregard for, Black lives in the USA. However, it was not until the 2014 murder of Mychal Brown by former Ferguson, MO officer Darren Wilson that the movement was brought to the forefront of the national discourse on racial inequities. Originated in the #blacklivesmatter hashtag, the movement quickly expanded to platforms outside social media. The BLM movement also symbolized a reaffirmation of Black lives in the USA despite systemic acts of discrimination perpetuated upon Black individuals and families. To date, BLM has 20 chapters across the USA and Canada. Since its inception, BLM has challenged anti-Black policy makers, facilitated the passage of critical legislation to support Black Americans, and, on a broader scale, arguably changed the national conversation on Blackness and the lived experiences of Black individuals [25].

The BLM movement has 13 principles that guide its philosophy and work for social change (Table 1). Several of its principles—namely, queer- and trans-affirming and empowering Black women—are firmly grounded in an intersectional understanding of the diversity of Black American experiences. Unlike previous social movements to redress systemic oppression, BLM places intersecting identities and transformative frameworks at the forefront of its epistemological grounding. Taken together, the principles put forth a more Africentric public health approach for understanding and empowering Black lives, families, and communities. Furthermore, the principles can be used to identify additional theories and frameworks for understanding Black health through Black lives.

**Table 1:** BLM principles

Principle	Description
Diversity	Acknowledging, respecting, and celebrating similarities and differences
Restorative justice	Working together vigorously for freedom and justice for Black people and, by extension, all people
Black affirming	Black people do not have to qualify their position
Globalism	Black people are part of a global Black family; acknowledgement of differential privileges within and across Black communities
Collective value	All Black Lives Matter
Transgender (trans) affirming	Embracing and making space for people of trans experience
Black women	Creating space for Black women free from misogyny, sexism, and male-centrism
Black villages	Supporting Black people as extended families and villages
Empathy	Engaging others with intent to learn about and connect with their contexts
Black families	Supporting family-friendly spaces for parents to fully participate with their children
Queer affirming	Fostering queer-affirming spaces free from heteronormativity
Age affirming	All people, regardless of age, have the capacity to lead and learn
Loving engagement	Embodying and practicing justice, liberation, and peace in interpersonal engagements

Adapted from Black Lives Matter (https://blacklivesmatter.com/what-we-believe/)

## A Proposed Alternative Approach for Black American Health

**Fig. 2:**
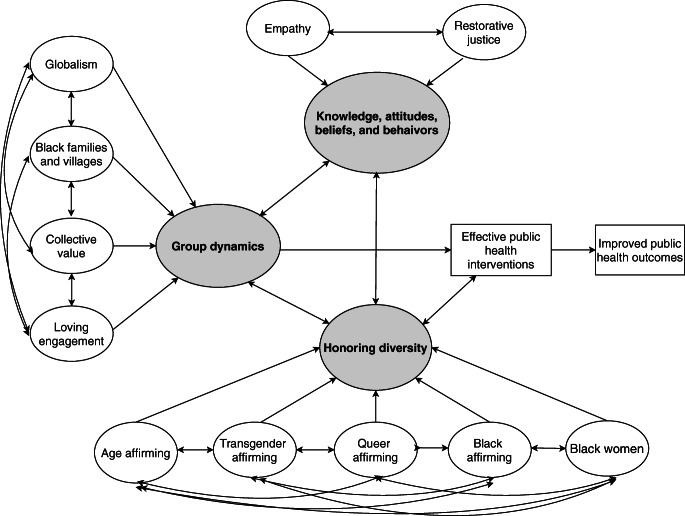
A proposed alternative approach to Black American health through the BLM principles. The unshaded circles represent the BLM principles and their interrelationships. The BLM principles are grouped into the three shaded constructs of honoring diversity, group dynamics, and attitudes and values. Together, the shaded areas, through the BLM principles, support the development and implementation of effective public health interventions for Black American communities, which may then positively impact public health outcomes

The alternative approach proposed in this article has three major constructs: (1) honoring diversity; (2) knowledge, attitudes, beliefs, and behaviors; and (3) group dynamics. This approach asserts that the concepts contribute to the development and implementation of effective public health interventions in Black populations, which then facilitate improved public health outcomes (Fig. 2). These constructs are bounded by the Africentric philosophy of “holism,” which refers to the interdependence between the whole person and the environment [26]. This concept is evident throughout the BLM principles. Specifically, the honoring of individual identities (e.g., age, sexual orientation, gender identity, and womanhood) is inextricably linked to the knowledge, attitudes, beliefs, and behaviors of Black Americans. These constructs, in turn, are interdependent with group dynamics (e.g., globalism, families and villages, restorative justice, and collective value). Together, these constructs help address the diversity in the Black American experience. Thus, through holism, the BLM principles work in concert to support intervention development and implementation, as well as positively impact health and social outcomes regarding Black American health.

The proposed approach to Black American health fits squarely within a networked conceptualization of the ecological systems theory while drawing on the strengths of the social determinants of health framework and theory of planned behavior. Not only can the model be tested for intervention development and implementation but it may also impact health and social outcomes for Black Americans. For instance, public health research can empirically test the degree to which the three major constructs contribute to intervention development and implementation.

### Honoring Diversity

The fundamental construct is honoring diversity, which relates to both individuals and microsystems in Neal and Neal’s [9] networked model of ecological systems. Because Black Americans are not a monolithic group, it is critical to begin by acknowledging the heterogeneity within these communities across the USA. Here, honoring diversity is conceptualized as acknowledging, embracing, and celebrating intersecting identities across Black American populations. It requires the inclusion of marginalized groups within Black populations not only as participants but also as citizen scientists in participatory research and action. In application, public health research might affirm transgender (trans) identities by creating an intellectual environment that allows Black people of trans experience to convey their lived experiences through oral histories to inform intervention development. Other approaches may affirm Black queer and trans identities through multicomponent interventions that integrate Africentric symbolism, spiritual rituals, and a celebration of their identities in traditional African cultures [27, 28]. Still, public health can—and should—acknowledge and address the contributions of slavery on Black American health, the harmful effects of geographical and cultural dislocation on Black American health, and how those factors work in tandem to disempower Black Americans in a capitalist economic framework [29].

Honoring diversity also includes embracing multiple affirming constructs simultaneously, denoted by double arrows in Fig. 2. This is an important feature because it highlights the intersecting identities present within Black American communities. Thus, maximizing efforts to honor diversity requires research and practice to consider not only an individual’s race but also one’s gender identity, sexual orientation, age, and social role simultaneously.

### Knowledge, Beliefs, Attitudes, and Behaviors

As Azjen [12] noted, one’s knowledge, beliefs, attitudes, and perceived behavioral control influence the extent to which individuals engage in healthy behaviors. Recently, studies have demonstrated the utility of assessing Black populations’ attitudes, perceived barriers, and perceived benefits of public health and medical interventions. Research based on the Theory of Planned Behavior found that, among Black American women, fear of condom negotiation was negatively associated with behavioral intent and condom use [30]. Still, later investigations suggest that barriers such as HIV stigma, lack of social support, and negative perceptions of healthcare providers are substantial barriers to engagement in the HIV continuum of care for Black individuals living with HIV [31, 32]. On the other hand, having social support, having a good relationship with healthcare providers, and reassurance about one’s health served as facilitating factors of care engagement [31, 33].

Despite the strengths of Azjen’s theory, other scholars have argued that the constructs within the theory must be placed in the proper cultural, social, and historical context to improve health outcomes among Black Americans. Using the Africentric principles noted by Randolph [26] and Banks, public health interventions for Black Americans should use multidimensional approaches that include storytelling and oral histories, as well as harmonious integration of emotional, cognitive, and behavioral aspects of the whole person. Such approaches may include acknowledging the health impacts of slavery and ongoing anti-Black policies and practices, honoring and integrating the diverse spiritual traditions that influence Black American health behaviors, and focusing on the process of changing health behavior and not only on the outcome itself [26].

Improving knowledge, attitudes, beliefs, and behaviors requires empathy and loving engagement. Rooted in the Nguni Bantu philosophy of *Ubuntu* (often translated as “I am because we are”), this construct invites public health research and practice to adopt a radical commitment to improving Black American health from an Africentric perspective. To effectively practice empathy and loving engagement, it is imperative for public health to draw from queer and Black feminist theories. For example, Black womanhood challenges the notion of the nuclear family (described in more detail below) because it is defined in part by Black American women’s experiences as “blood mothers, othermothers, and community othermothers.” [34]^(p. 554)^ In this vein, understanding the critical role of Black womanhood in Black American communities can enhance initiatives to improve health outcomes. By also anchoring research and interventions in the understanding of identities such and queer and trans identities in the traditional African context [27, 28], public health can foster empathy and promote loving engagement among Black Americans regarding health behaviors, which may ultimately change Black Americans’ health-related knowledge, attitudes, beliefs, and behaviors. Thus, constructs such as empathy and loving engagement necessitate expanding the field of public health to effectuate community-oriented interventions that include Black community members as integral partners in development, implementation, evaluation, and sustainment.

### Group Dynamics

Group dynamics refers to behavioral and psychological processes occurring within or between social groups [35, 36]. In terms of the ecological systems theory, group dynamics include both microsystems and mesosystemic interactions. Distally, it includes mechanisms that support upstream (or exosystemic) efforts, such as policy changes and widespread practice changes. In the proposed approach, group dynamics accounts for interpersonal behavioral and psychological mechanisms across the social ecology. In sum, the construct is an amalgamation of community-oriented actions rooted in empathy, love, and diversity, in which the liberty of the collective unit is the sum of individual liberties.

At its most basic ecological level, the approach promotes positive psychological and behavioral change by promoting Black families and villages. Contrary to Western conceptualizations of the family unit, an Africentric perspective views family as far more extensive than the nuclear family. In Black American families, as Davis and Davis note [29], the horrors of families being ripped apart due to slavery meant that Black families could not conform perfectly to the Eurocentric model of the nuclear family. As such, public health research and practice must account for the fact that “family” often extends beyond the nuclear or household unit. It also requires public health research and practice to examine epistemological assumptions about Black families and villages while challenging patriarchal norms. This charge can be kept by integrating Black feminist and queer theories into understanding the realities, processes, and health behaviors in Black American subpopulations.

Another key construct is restorative justice. To understand its positioning in the overall proposed approach, we turn to the BLM movement in its framing of restorative justice:

We work vigorously for freedom and justice for Black people and, by extension, all

people. We intentionally build and nurture a beloved community that is bonded together

through a beautiful struggle that is restorative, not depleting.^25, para. 7^

Given the movement’s framing, it stands to reason that restorative justice extends beyond areas of criminal justice. Rather, it includes areas such as psychosocial, behavioral, and environmental determinants of health, education, and chronic disease. To be sure, research is clear regarding the effectiveness and utility of restorative justice efforts in diverse settings. Recent work such as the Young Women’s Empowerment Project [37] has been effective in enhancing resistance to harm, re-centering understanding of empowered safety, and reorienting attitudes and beliefs in self-care to address harm. Other work has demonstrated positive effects in improving student outcomes [38, 39], suggesting that youth who participate in restorative justice interventions are more likely to receive disciplinary referrals and suspensions while emerging as participatory leaders.

To address Black health from a public health perspective, it is necessary to reorient how the field understands restorative justice. Using the aforementioned framing, public health interventions can capitalize on the knowledge within Black communities by including them as community experts. Additionally, public health research and practice can build capacity in Black communities to conduct community assessments, mobilize and plan action, implement and evaluate action plans, and sustain their efforts. Although there are many ways that public health efforts can integrate restorative justice, the core component of the construct is to develop and implement public health interventions that bring diverse Black populations together in ways that also promote empathy and loving engagement.

Considering the implications of intervention development in Black families, one should also understand the concept of Black villages. According to the BLM movement, Black villages have two goals: (a) to contest the Western-dominated conceptualization of family structure (i.e., two-parent nuclear family) and (b) to support one another as part of extended families “*to the degree that parents and children are comfortable”* (emphasis added). These goals suggest that, based on the networked ecological systems theory [9], it is important to investigate the impact of multiple mesosystemic interactions from an Africentric perspective.

A fundamental assumption is collective value—that is, *all* Black Lives Matter. All Black lives across the globe should be honored, respected, and celebrated regardless of identity, privilege, citizenship status, or worldviews. Without this core assumption, one cannot fully examine group dynamics in Black populations. And, by extension, this proposed approach would be inconsistent with itself. A corollary to collective value is globalism or the position that each Black individual is a member of a global, diasporic Black family. Within the position of globalism lies the understanding that diversity of experience exists across Black subpopulations such that some Black communities may experience privileges that are foreign to others. Because privilege is intersectional, it stands to reason that factors such as socioeconomic status, gender identity, and sexual orientation may place some Black subgroups in positions of relative privilege than others. Thus, systematic investigations in the role of constructs such as collective value and globalism not only contribute to identify effective interventions but they also support the Black-affirming nature of the alternative approach proposed here.

## Limitations, Considerations, and Future Directions

As argued in this article, the proposed approach may provide a more refined approach for addressing health outcomes in Black American populations. By concentrating efforts on honoring diversity, promoting prosocial attitudes and values, and fostering group dynamics, public health research and practice can support more effective interventions, thus yielding improved public health outcomes for Black populations. Furthermore, the proposition put forth in this article allows public health research to develop instruments to measure more specific constructs such as restorative justice through an Africentric lens. A critical feature is its focus on affirming Black lives—not only in research and practice but also in theory development and testing.

Nevertheless, many considerations must be acknowledged. First, it is important to note that the proposed approach is applicable to Black populations in the USA; as such, it may not hold for other Black populations across the world. Furthermore, this approach is not intended to co-op the important work of the BLM movement. Rather, it provides an avenue to anchor public health research and practice in a conceptual understanding of the collective lived experiences, core values, and ideals of Black people in the USA from an Africentric perspective.

Additionally, many constructs are interrelated, and empirical research is needed to investigate the strength of those relationships across multiple communities of the African Diaspora. It must also be noted that the proposed approach in this article is not a panacea for conceptualizing Black American health for all Black populations across the African Diaspora. Rather, it provides an alternative approach by which rigorous, culturally responsive scientific investigations can be conducted. Furthermore, the two-dimensional nature shown in Fig. 2 suggests that constructs of honoring diversity and knowledge, attitudes, beliefs, and behaviors mediate the relationship between group dynamics and effective public health interventions. That is not the case. A correct interpretation would require a three-dimensional structure depicting the unique contributions of each major construct (i.e., group dynamics; knowledge, attitudes, beliefs, and behaviors; honoring diversity) on developing and implementing effective public health interventions. Thus, future work can empirically test the relative contributions of each constructs on public health outcomes for Black communities. Additionally, the proposed approach may afford public health research and practice a basis for creating culturally responsive instruments to measure implementation fidelity of interventions from a more Africentric perspective.

Considerations notwithstanding, the proposed alternative conceptualization of Black American health is rooted in the principles of the BLM movement and Africentrism and informed by the ecological systems theory and social determinants of health model. Unlike most public health theories and frameworks, this approach integrates Africentric thought to inform the development, implementation, and evaluation of public health interventions regarding Black American health. In addition, it shows the interactive and iterative nature of core principles in understanding Black health and well-being. First, it demonstrates that each major construct is networked with the others. Second, within each major construct lie subconstructs that intentionally yield themselves to robust measures of core dimensions. For example, measuring collective value may include dimensions such as self and perceived identity, as well as situational value in law enforcement, educational institutions, and social structures. Finally, it provides linkages between theoretical and methodological advances in improving population-level health outcomes for Black communities.

The approach presented in this article calls for public health to re-examine its prevailing ontology, epistemology, and axiology regarding Black American health. Embracing a more critical, Africentric perspective requires public health to honor the collective reality of Black American health that has been damaged by racial trauma, economic capitalism, and social silencing. It also calls for the field to adopt alternative means for understanding that collective reality that align with Africentric values, such as spiritual traditions through storytelling and oral histories. As an alternative conceptualization of improving Black American health outcomes, I hope the approach presented here offers public health researchers a useful manner of thinking about and addressing Black American health and that this article serves as a first step in further conceptual refinement and intervention development.

## Footnotes

1 Although there is disagreement on whether to use Black or African American, I will use the Black identifier throughout this article to underscore that some people of African descent living in the USA are descendants of Africans from across the transatlantic slave trade.
