# Correction to: Black Lives Matter Principles as an Africentric Approach to Improving Black American Health

**DOI:** 10.1007/s40615-020-00858-9

**Published:** 2020-09-01

**Authors:** Kaston D. Anderson-Carpenter

**Affiliations:** Michigan State University, East Lansing, MI USA

**Correction to: Journal of Racial and Ethnic Health Disparities**

10.1007/s40615-020-00845-0

Readers should note that the graphic for Fig. 2 in this article is not the final version of the figure that was intended by the author.

The correct version of Fig. 2 is provided below.



